# Finite Element Modeling of the Dynamic Properties of Composite Steel–Polymer Concrete Beams

**DOI:** 10.3390/ma13071630

**Published:** 2020-04-01

**Authors:** Paweł Dunaj, Stefan Berczyński, Marcin Chodźko, Beata Niesterowicz

**Affiliations:** Department of Mechanical Engineering and Mechatronics, West Pomeranian University of Technology, Szczecin, al. Piastów 19, 71-310 Szczecin, Poland; stefan.berczynski@zut.edu.pl (S.B.); beata.niesterowicz@zut.edu.pl (B.N.)

**Keywords:** finite element modeling, polymer concrete, composite beams, modal analysis, structural component dynamics

## Abstract

This paper presents a method for modeling the dynamic properties of steel–polymer concrete beams, the basic structural components of machine tools, assembly lines, vibratory machines, and other structures subjected to time-varying loads during operation. The presented method of modeling steel–polymer concrete beams was developed using the finite element method. Three models of beams differing in cross-sectional dimensions showed high agreement with experimental data: relative error in the case of natural frequencies did not exceed 5% (2.2% on average), the models were characterized by the full agreement of mode shapes and high agreement of frequency response functions with the results of experimental tests. Additionally, the developed beam models supported the reliable description of complex structures, as demonstrated on a spatial frame, obtaining a relative error for natural frequencies of less than 3% (on average 1.7%). Full agreement with the mode shapes and high agreement with the frequency response functions were achieved in the analyzed frequency range.

## 1. Introduction

Successful diagnosis of the static and dynamic properties of composite structural elements requires the development of a reliable model as a tool to assist engineers in making design decisions. One such composite structural element is the steel–concrete or steel–polymer-concrete beam. Despite the extensive literature on the modeling of the static properties of such structures [1,2,3,4,5,6,7,8,9], few publications deal with the modeling of their dynamic properties. This may indicate a more developed understanding of the aspects related to their static properties in relation to their dynamic properties.

In [10], the authors presented continuous models enabling the description of free vibrations in composite steel and concrete beams. The analyzed beams consisted of a steel I-section beam connected with a concrete slab using stud connectors. The paper presents two continuous models based on the Euler–Bernoulli beam theory and one model built according to the Timoshenko beam theory. The Euler–Bernoulli model takes into account the elasticity of the stud connection while the Timoshenko model treats it as rigid, taking into account the stiffness of the junction. The developed models were subjected to experimental verification, from which it was found that the Timoshenko beam theory model best described the dynamic properties of the considered beam, with a relative error for the natural vibration frequencies on average of 3.3% and not exceeding 5.4%. In the case of the classical Euler–Bernoulli beam theory model, the relative errors for the natural vibration frequencies for the higher mode shapes even reached 50%.

In [11], the authors presented composite steel–concrete beam models constructed according to Euler–Bernoulli and Timoshenko beam theories, allowing descriptions of the dynamic properties, i.e., natural frequencies and mode shapes. By experimentally verifying the results obtained on the basis of the model analyses, the authors obtained full compliance for the first seven mode shapes in both models with relative errors not exceeding 29.2% (on average 15.4%) for the Euler–Bernoulli model, and not exceeding 9.1% (on average 4.9%) for the Timoshenko model.

In [12], the authors presented analytical prediction models for the elastic behavior and first eigenfrequency of non-prismatic composite steel–concrete beams with non-uniform shear connector arrangements. That approach was based on the sixth and second order differential equations used to define matrix equations for a finite number of linearized composite beam segments. The analytical models were validated using numerical FEM results of a simple supported tapered composite beam. The results showed a good agreement between the eigenfrequencies obtained using the proposed analytical model and FEM. The proposed analytical model underestimated the first eigenfrequency by 0.3% on average. However, the authors did not conduct an experimental verification of the results of the dynamic properties obtained from the developed model.

In [13], a hybrid finite element–statistical energy analysis (FE–SEA) method was used to investigate the structure-borne noise in a steel–concrete railway bridge, which, in a structural sense, can be treated as a composite beam. A hybrid FE–SEA method was introduced in which FE was used to model the concrete deck and SEA was used to model the steel girders. The established model allowed the computation of the vibration and noise of the composite railway bridge. The proposed method was verified by comparing its predictions with field measurements. The authors concluded that the hybrid FE–SEA model had a good balance between accuracy and efficiency when computing the vibration and noise of composite structures with a variety of modal densities. However, the authors did not provide a quantitative measure of the accuracy of the model.

In [14], the authors presented a method for modeling the dynamic properties of a milling machine column consisting of a cast iron frame, elastomer and concrete. The model was also developed using FEM. In the first stage, the authors constructed a model of the cast iron frame which fully reflected its geometry; rounding, chamfering, wall inclination and ribbing were taken into account. Then, the natural frequencies and mode shapes were determined for an unconstrained model. The obtained results were compared with those from an experimental study, resulting in a relative error for natural frequencies up to 10%. Due to the fact that the model of the cast iron frame itself was biased with significant inaccuracy in representing its dynamic properties, it was decided thar a model with a simplified construction would be developed. For this purpose, the geometric model was reduced to a beam with a 1:5 scale square cross-section in relation to the real structure. Experimental verification of the simplified cast iron structure showed a relative error for natural frequencies below 4.3%. Then, experimental verification of the model of a beam filled with elastomer and concrete showed a relative error for natural frequencies below 2.3% (on average 0.9%).

The aforementioned works indicate that accurate computational analysis is crucial in designing composite machine components. However, the literature barely mentions the experimental verification of frequency response functions to describe the dynamic properties across wider frequency ranges. The agreement of individual natural frequencies between the model and experiment is just one and not even the most important aspect of validating modeling results. The more important factors are the agreement of the mode shapes for individual natural frequencies (structural agreement) with the frequency response functions in a given frequency range.

This paper presents a method for the finite element modeling (FEM) of the dynamic properties (natural frequencies, mode shapes and frequency response functions) of composite structural components consisting of a hollow steel profile filled with a highly heterogenous polymer concrete. The process of modeling the dynamic properties of the described beams required appropriate simplifying assumptions. These assumptions determined the structure of the model, the methods of determining its parameters, and the choice of an appropriate validation method (e.g., the assumption of the linearity of the stiffness of the considered beam allows for validation using experimental modal analysis). Assumptions made during modeling resulted from a study on the modeled object based on a series of experiments. The experiments were carried out in two stages: the first aimed at determining the material properties of the steel and polymer concrete, and the second aimed at providing information about the dynamic properties of the object. The presented method was verified for beams with a variety of cross-sectional dimensions, as well as a composite frame composed of such beams. In this paper, it has been proven in experimental, analytical and numerical studies that highly heterogeneous polymer concrete can be modeled using a linear isotropic model of the material, provided that it is present in the composite steel–polymer concrete structure.

The structure of the paper is as follows: in Section 2, the steel–polymer concrete beam concept is presented. Next, experimental studies to determine the static and dynamic properties of the analyzed beam are conducted. In Section 3, a finite element model of the beam is established. The natural frequencies, mode shapes and frequency response functions obtained from the proposed model are compared with continuous models (the Timoshenko model for bending and shear modes, and the Saint-Venant model for torsional modes) and, subsequently, experimentally verified. Next, the model of a frame consisting of steel–polymer concrete beams is built and experimentally verified. In Section 4, a discussion of the results obtained is provided. Section 5 contains the final conclusions that summarize the most important achievements of the article.

## 2. Materials and Methods

### 2.1. Steel–Polymer Concrete Beam Concept

The concept of the presented beam is based on the synergic use of steel (ensuring the assumed stiffness of the structure) and polymer concrete (increasing its ability to dissipate vibration energy). When properly arranged and appropriately filled with polymer concrete, the beams allow the shaping of the dynamic properties of larger structures [15,16]. Polymer concrete was selected on the basis of previous studies [17,18] for its ability to significantly increase the energy dissipation of steel structure, and a small shrinkage—after filling the steel profile, the polymer concrete adhered to its internal surface.

Selected polymer concrete consisted of an epoxy resin mixed with different grades of mineral filling. Taking the grain size as the dividing criteria, mineral fillings can be categorized as follows: ash, a fine fraction (consisting mainly of sand) of grain size 0.25–2 mm, a medium fraction of grain size 2–10 mm and a coarse fraction of grain size 8–16 mm. The coarse and medium fractions comprised mainly irregular shaped gravel. The proportion of the mass (percentage) of the individual fractions in the polymer concrete are presented in Table 1.

**Figure 1:**
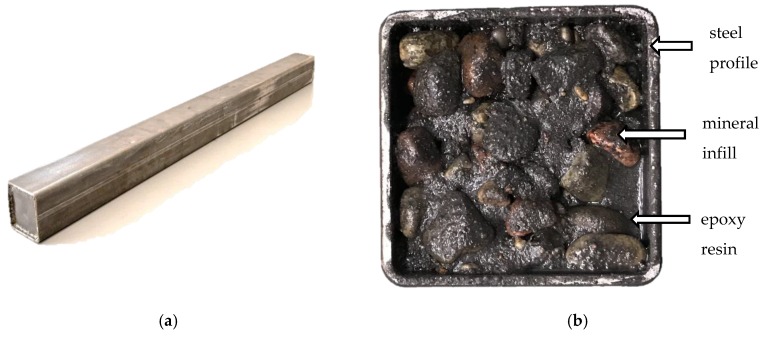
Steel–polymer concrete beam (**a**) general view; (**b**) cross-section view.

The presented polymer concrete was poured into the steel profiles and fully vibrated. In this research, 1 m lengths of three different square cross-sectional steel profiles filled with polymer concrete were used: (i) 140 mm × 140 mm, 6 mm wall, (ii) 70 mm × 70 mm, 3 mm wall, and (iii) 50 mm × 50 mm, 2 mm wall. All the specimens were cured in the room conditions (at 23 °C and 50% relative humidity) for 72 h. The steel–polymer concrete beam is presented in Figure 1.

### 2.2. Static Tests

**Figure 2:**
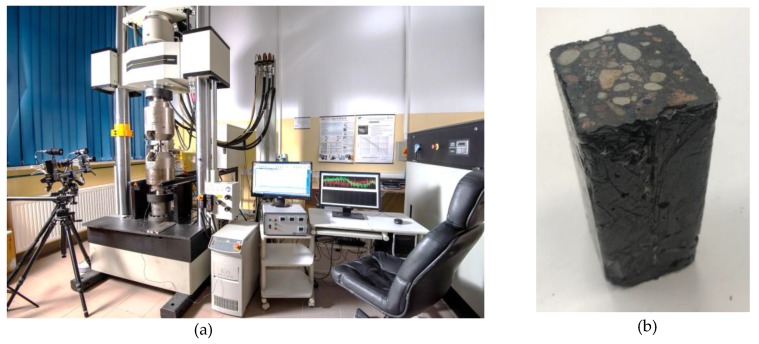
Test stand for compression tests (**a**) Instron 8850 press; (**b**) tested polymer concrete sample.

In the first stage of the experiment, the material parameters (modulus of elasticity, Poisson’s ratio, density) of the steel and polymer concrete were determined. Cuboidal samples of steel (50 mm × 50 mm × 110 mm) and polymer concrete (45 mm × 45 mm × 110 mm) were subject to compression tests using an Instron 8850 press (Instron, Norwood, MA, USA) operating in an air-conditioned laboratory at 23 °C and 50% relative humidity. The press was equipped with a class 0.5 measuring head with a range up to 250 kN and a class 0.2 Instron 2620-603 (Instron, Norwood, MA, USA) tactile extensometer with a 25 mm and 12.5 mm measuring range (±1 mm). The samples were conditioned in the laboratory for 72 h prior to testing [19]. The test stand is presented in Figure 2.

### 2.3. Dynamic Tests

**Figure 3:**
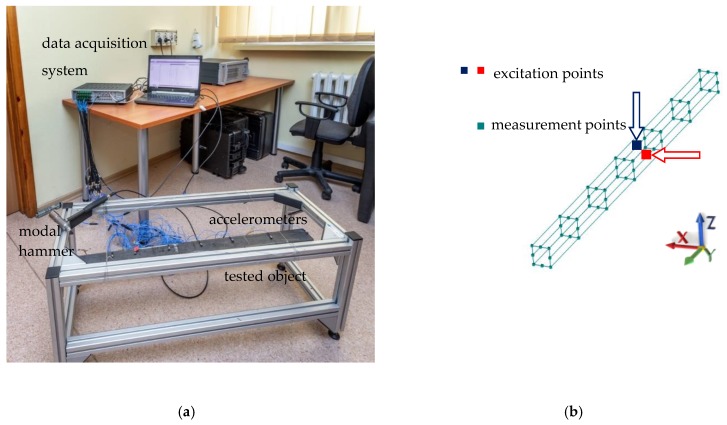
The test stand for the dynamic properties of the steel–polymer concrete beam (**a**) test stand; (**b**) measurement points arrangement.

The second stage of the experimental investigations consisted of a modal analysis. An impact test was carried out for the 1 m long 70 mm × 70 mm × 3 mm steel–polymer concrete beam. The tested beam was suspended at each end using steel ropes to minimize the influence of the stand. A diagram of the test stand is shown in Figure 3. Vibrations in the beam were excited in two mutually perpendicular directions (+X and −Z, Figure 3) using a PCB 086C0 modal hammer (PCB Piezotronics, Depew, NY, USA). Measurements of the response were taken at 56 points using PCB triaxial ICP sensors, model 356A01 (PCB Piezotronics, Depew, New York, NY, USA). The signals were acquired using a Scadas Mobile Vibco analyzer (Siemens, Munich, Germany). Selected parameters of the signal acquisitions are presented in Table 2 [19].

**Figure 4:**
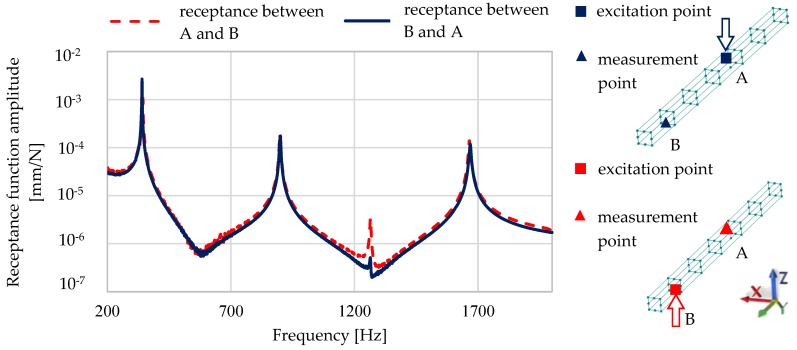
Comparison of the receptance functions determined to verify the Maxwell reciprocal theorem.

The basic assumptions of the modal analysis (Maxwell reciprocal theorem—Figure 4, damping proportionality, system linearity) were verified before the final investigations.

The verification of the assumptions of the modal analysis was followed by final research, including the determination of 112 frequency response functions. The estimation of the modal model parameters was performed using the Polymax algorithm [20,21]. Due to the real and complex mode shape identity, real mode shapes were used. Using the Model Assurance Criterion (MAC) to determine the orthogonality of the mode shape vectors, the estimated modal model was validated by eliminating the interdependent vectors of the mode shapes (a limit value of 10% was adopted) [22,23].

### 2.4. Experimental Test Results

The experimental test results, i.e., material properties with standard uncertainties supplemented by the value of the loss factor determined on the basis of dynamic tests (using the half power method), are summarized in Table 3.

**Figure 5:**
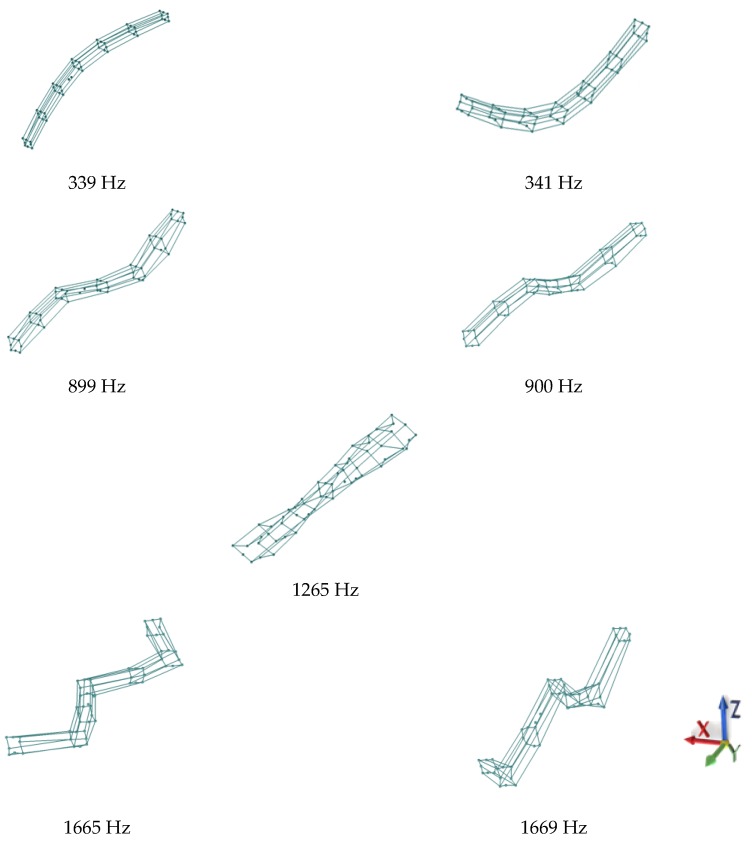
Experimentally determined mode shapes of the steel–polymer concrete beam.

The parameters of the modal model in the form of natural frequencies and corresponding frequencies of modal damping are presented in Table 4. The natural frequencies are shown in Figure 5.

The results of the experimental study showed that:

- The steel–polymer concrete beam fulfilled the basic assumptions of the modal analysis, which validates its use in the assessment of its dynamic properties;
- The steel–polymer concrete beam is characterized by the close identity between the complex and real mode shapes, which indicates the proportional damping of the beam;
- Despite the fact that the steel–polymer concrete beam was made of materials with significantly different properties, the experimental mode shapes were similar to those of a beam made of homogeneous material, which indicates a lack of mutual displacements between the steel profile and polymer concrete filling.

## 3. Modeling of the Steel–Polymer Concrete Beams

### 3.1. Model Assumptions

**Figure 6:**
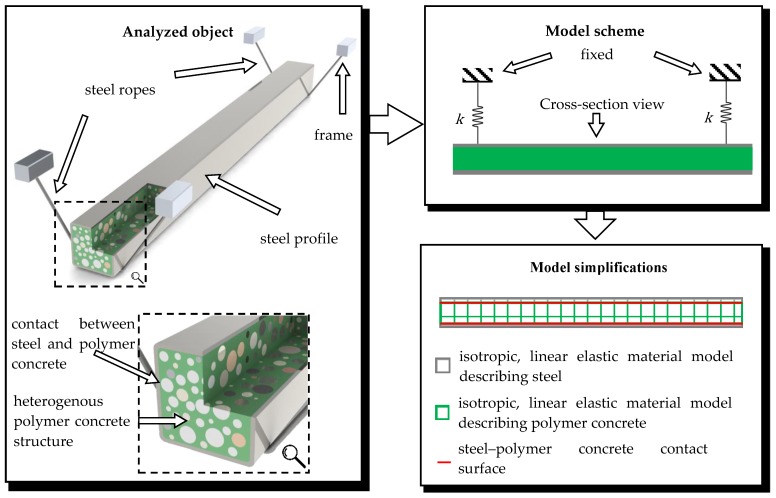
Simplifications applied for the mathematical model of the steel–polymer concrete beam.

Based on the experimental study, certain simplifications were used in order to build the FEM model. A schematic representation of the adopted simplifications of the model is shown in Figure 6.

It was assumed that the suspension method of the tested beam during the experimental studies would have a negligible influence on the dynamic properties. It was therefore assumed that the calculations could be carried out as if for an unconstrained (free–free) model.

Another assumption was made for the model of the material used to describe the dynamic properties of the polymer concrete. Despite its heterogeneous structure, it was assumed that in the studied frequency range and in the case of displacements not exceeding 0.5 mm (the maximum amplitude of vibrations in machine tool bodies usually do not exceed this value [24,25]), the polymer concrete can be represented using a linear elastic isotropic model. The basis for this assumption was seen in the experimental results bia the fulfilment of the Maxwell reciprocal theorem, linear stiffness, and symmetry of the mode shapes, determined from the impact test. The material properties were defined on the basis of the experimental results presented in Table 3.

In the next step, a mathematical model of damping was selected to describe the ability of the steel–polymer concrete beam to dissipate the vibration energy. Analysis concerned Rayleigh damping [26,27] and the structural damping model expressed as complex stiffness [28,29]. The differences following these models turned out to be insignificant, with a detailed analysis presented in a previous work [30]. On this basis, a structural damping model was used to describe the damping properties of the modeled beam, according to which the damping matrix C can be expressed as:

(1) C=jηK

where K is the stiffness matrix, j is the imaginary unit and η is the loss factor.

It was then assumed that the contact between the steel profile and the polymer concrete filling occurs over the entire internal surface of the steel profile. In addition, the adhesion forces prevent tangential displacement within the contact area of the materials. Hence, the contact of the steel profile with the composite filling was modeled via the coincidence of nodes. The application of this model was dictated by the analogy between the analyzed beam and concrete filled steel tubes (CFST) used in civil engineering. Research on CFST [31,32,33,34,35,36,37] shows that the steel profile and concrete filling behave together as a structure made of a uniform material. Additionally, research shows that steel pipes filled with concrete are subject to Saint-Venant’s Principle [38].

### 3.2. Finite Element Model of A Steel–Polymer Concrete Beam

**Figure 7:**
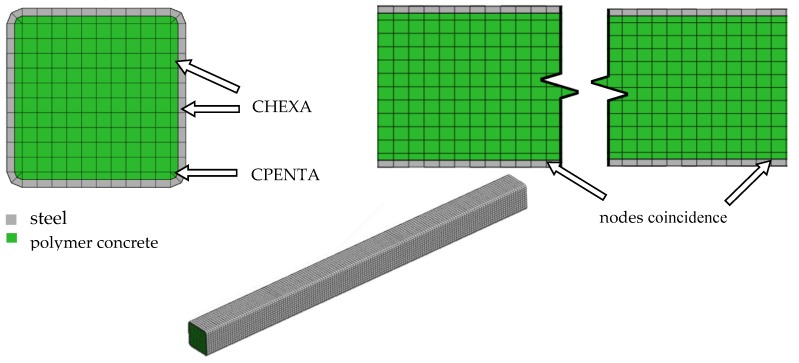
A discrete model of the steel–polymer concrete beam.

Based on the assumptions made, the geometric model was discretized using a Midas NFX 2018 R1 preprocessor (Midas Information Technology Co. Ltd., Seongnam, Korea) [39]. The calculation area was divided into CHEXA eight-node six-sided isoparametric finite elements, and CPENTA six-node five-sided isoparametric elements. The applied finite elements were characterized by linear shape functions and three translation degrees of freedom in each node. The process of discretization was based on a structural grid. The construction of the grid was supported by the analysis of its quality, considering the coefficients of aspect ratio and skewness. In total, the developed model consisted of 19,600 elements and had 21,985 degrees of freedom; the discrete model is shown in Figure 7.

Then, the natural frequencies and corresponding mode shapes were determined for the unconstrained model of the beam. This task was realized by solving the eigenproblem in the following way:

(2) $$(K-{\omega_{n}}^{2}M)\;\Phi_{n}=0$$

where: M is the the inertia matrix of the model, $\omega_{n}$ is the n-th value of the natural frequency and $\Phi_{n}$ is the n-th mode shape vector. The calculations were performed using Nastran Solver (SOL103).

### 3.3. Timoshenko Beam Model of a Steel–Polymer Concrete Beam

In order to compare the accuracy of the established discrete FEM model, a continuous mathematical model (used to model this type of structure in most of the research works presented in the introduction) was defined. The model is based on the Timoshenko beam theory for bending and shear deformation and Saint-Venant’s theory for torsional vibration. In order to determine the coefficients of the steel–polymer concrete beam equations, Hamilton’s principle was applied. Assuming that the Timoshenko model takes into account flexural, shear and longitudinal deformations, the equation for a beam with free vibrations can be determined as:

The elements of this equation may be expressed as

(3) $$\begin{array}{c} \left(E_{1}I_{1}+E_{2}I_{2}\right)\frac{\partial^{4}w}{\partial x^{4}}+\left(\rho_{1}A_{1}+\rho_{2}A_{2}\right)\frac{\partial^{2}w}{\partial t^{2}}-\left(\rho_{1}I_{1}+\rho_{2}I_{2}\right)\left(1+\frac{\left(E_{1}I_{1}+E_{2}I_{2}\right)\left(\rho_{1}A_{1}+\rho_{2}A_{2}\right)}{k\left(A_{1}G_{1}+A_{2}G_{2}\right)}\right)\frac{\partial^{4}w}{\partial x^{2}\partial t^{2}}\\ +\frac{\left(\rho_{1}I_{1}+\rho_{2}I_{2}\right)\left(\rho_{1}A_{1}+\rho_{2}A_{2}\right)}{k\left(A_{1}G_{1}+A_{2}G_{2}\right)}\frac{\partial^{4}w}{\partial t^{4}}=0 \end{array}$$

The elements of this equation may be expressed as ±*λ*_1_*j*, ±*λ*_2_. The general solution for natural vibration analysis takes the following form:

(4) W(x,t)=w(x) q(t)

After separating the variables, taking into account boundary conditions appropriate for a free–free beam resulting from the fact that the bending moments and shear forces at both ends of the beam equal zero and substituting the boundary conditions at both ends of the beam (x=0 and x=L), Equation (5) is obtained.

(5) $$w\left(x\right)=C_{n}\left(\frac{{\lambda_{2}}^{3}}{{\lambda_{1}}^{3}}sinh\lambda_{1}x+sin\lambda_{2}x+\frac{sin\lambda_{2}x-sinh\lambda_{1}x}{cosh\lambda_{1}x-cos\lambda_{2}x}\left(\frac{{\lambda_{2}}^{2}}{{\lambda_{1}}^{2}}cosh\lambda_{1}x+cos\lambda_{2}x\right)\right)$$

After defining the matrix of coefficients of integral constants, the characteristic equation can be finally presented in the form:

(6) $$w\left(x\right)=C_{n}\left(\frac{{\lambda_{2}}^{3}}{{\lambda_{1}}^{3}}sinh\lambda_{1}x+sin\lambda_{2}x+\frac{sin\lambda_{2}x-sinh\lambda_{1}x}{cosh\lambda_{1}x-cos\lambda_{2}x}\left(\frac{{\lambda_{2}}^{2}}{{\lambda_{1}}^{2}}cosh\lambda_{1}x+cos\lambda_{2}x\right)\right)$$

The approximate solution for this equation is found with these elements in the following form:

(7) $$\lambda_{m}=\left(2m-1\right)\frac{\pi }{2}$$

The natural bending and shear vibration frequencies were determined from the following equation:

(8) $$\frac{\left(\rho_{1}I_{1}+\rho_{2}I_{2}\right)}{k\left(A_{1}G_{1}+A_{2}G_{2}\right)}\omega_{n}^{4}-\left(1+\frac{{\lambda_{m}}^{2}\left(\rho_{1}I_{1}+\rho_{2}I_{2}\right)}{L^{2}\left(\rho_{1}A_{1}+\rho_{2}A_{2}\right)}+\frac{{\lambda_{m}}^{2}\left(E_{1}I_{1}+E_{2}I_{2}\right)}{L^{2}k\left(A_{1}G_{1}+A_{2}G_{2}\right)}\right)\omega_{n}^{2}+\frac{{\lambda_{m}}^{4}\left(E_{1}I_{1}+E_{2}I_{2}\right)}{L^{4}\left(\rho_{1}A_{1}+\rho_{2}A_{2}\right)}=0$$

Based on Saint-Venant’s theory and Hamilton’s principle, the following equation of motion to determine torsional frequencies is:

(9) $$\frac{\partial^{2}\theta }{\partial x^{2}}-\frac{1}{C}\frac{\partial^{2}\theta }{\partial t^{2}}=0\;\mathrm{with}\;C=\sqrt{\frac{\left(G_{1}K_{1}+G_{2}K_{2}\right)}{\left(\rho_{1}I_{p1}+\rho_{2}I_{p2}\right)}}$$

where $K_{i}$ is a torsional parameter, which, for a non-circular hollow square cross-section, equals:

(10) $$K_{i}=0.1408\left({H_{i}}^{2}-{h_{i}}^{2}\right)$$

After the application of boundary conditions adequate for both free ends, we get torsional natural frequencies in the form:

(11) $$\omega =\frac{m\pi }{L}C.$$

### 3.4. Results Comparison

A comparison of the natural frequencies determined in the model analysis, with the results of the experimental studies is presented in Table 5. The agreement between the natural frequencies in the FEM and continuous model of the beam with those determined experimentally was presented as the relative error δ, defined in the following way:

(12) $$\delta =\left|\frac{\omega_{exp}-\omega_{model}}{\omega_{exp}}\right|\times 100$$

where $\omega_{exp}$ is the experimentally determined natural frequency, $\omega_{model}$ is the natural frequency calculated for the FEM model and for the continuous model (C).

**Figure 8:**
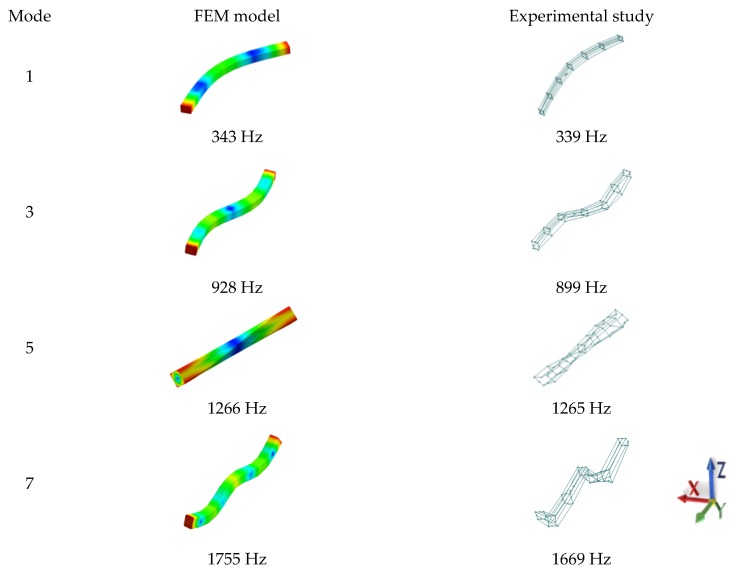
A comparison of selected calculated and experimentally verified mode shapes of the steel–polymer concrete.

**Figure 9:**
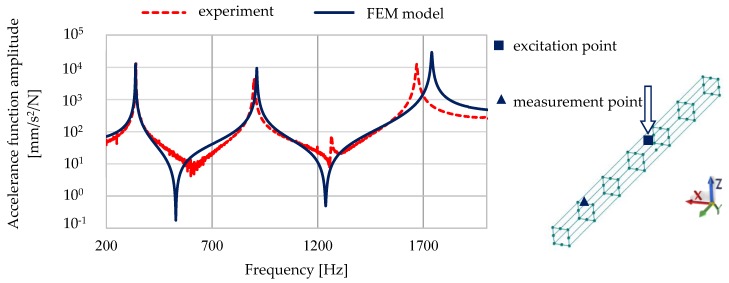
Experimental verification of the accelerance function for the model of a steel–polymer concrete beam with identified parameters.

A comparison of the calculated and experimentally verified mode shapes is presented in Figure 8. Figure 9 presents experimental verification of the accelerance function for the model of a steel–polymer concrete beam with identified parameters.

### 3.5. Model Validation

The last stage of building the model of a steel–polymer concrete beam was the validation of the modeling procedure. Using the developed modeling procedure and the values of the identified model parameters, two variants of 1 m long unconstrained steel–polymer concrete beams, (1) 50 mm × 50 mm, 2 mm wall thickness, and (2) 140 mm × 140 mm, 6 mm wall thickness, were modeled. Then, the results of the model analyses were compared with the results of the experimental tests.

Table 6 shows the natural frequencies of the two types of 1 m long beam (50 mm × 50 mm and 140 mm × 140 mm), determined on the basis of the models and experiments analogous to those presented in Section 3. Five mode shapes were identified in the studied frequency range (30–2000 Hz).

**Figure 10:**
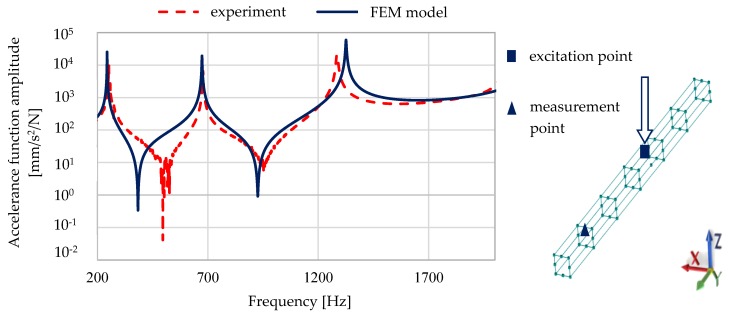
Comparison of calculated and experimentally determined accelerance functions for a 50 mm × 50 mm steel–polymer concrete beam.

**Figure 11:**
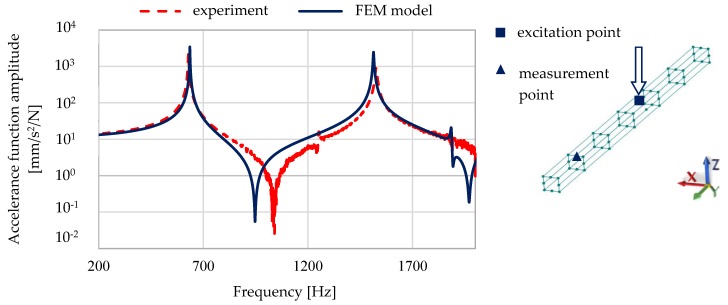
Comparison of calculated and experimentally determined accelerance functions for a 140 mm × 140 mm steel–polymer concrete beam.

Figure 10 and Figure 11 show a comparison of selected accelerance functions determined for a 50 mm × 50 mm and 140 mm × 140 mm steel–polymer concrete beam on the basis of FEM models and experiments.

### 3.6. An Example of Using A Steel–Polymer Concrete Beam Model

#### 3.6.1. Finite Element Model of Steel–Polymer Concrete Frame

**Figure 12:**
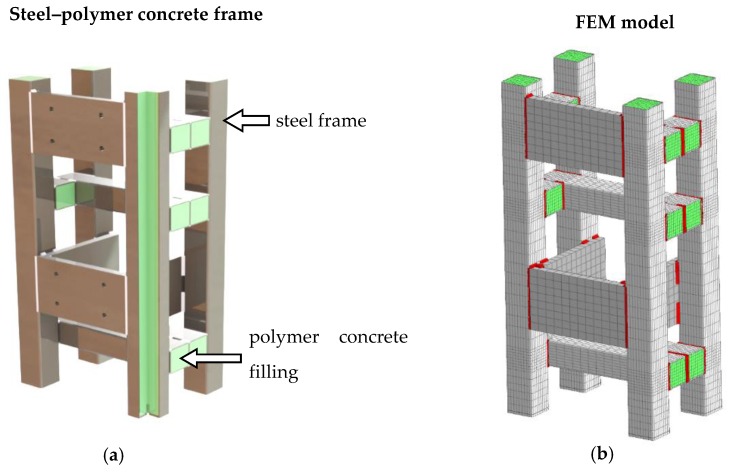
A frame consisting of steel–polymer concrete beams (**a**) geometric model; (**b**) finite element model.

In order to show the usefulness of the established model of a steel–polymer concrete beam, a model was built of a frame consisting of 50 mm × 50 mm steel–polymer concrete beams (Figure 12).

The developed unconstrained FEM model of the steel–polymer concrete structure consisted of 60,584 finite elements (CHEXA and CPENTA) and 225,384 degrees of freedom [19]. The eigenproblem of this frame was solved and the frequency response functions determined.

#### 3.6.2. Experimental Verification of the Finite Element Model

The obtained results were then experimentally verified. Experimental tests of the real object were carried out using an impact test and the stand is presented in Section 3. The point of application of force was selected to excite the mode shapes as much as possible within the studied frequency range (30–1250 Hz). Excitation was performed in three orthogonal directions. The response of the actual construction to the given excitation was measured at 260 points. The distribution of these points was selected on the basis of the analysis of the mode shapes obtained for the FEM model. As a result of the experiment, 780 frequency response functions were obtained. The modal model was built analogously to the one presented in Section 3. A comparison of the values determined by calculation and experimentally is presented in Table 7.

**Figure 13:**
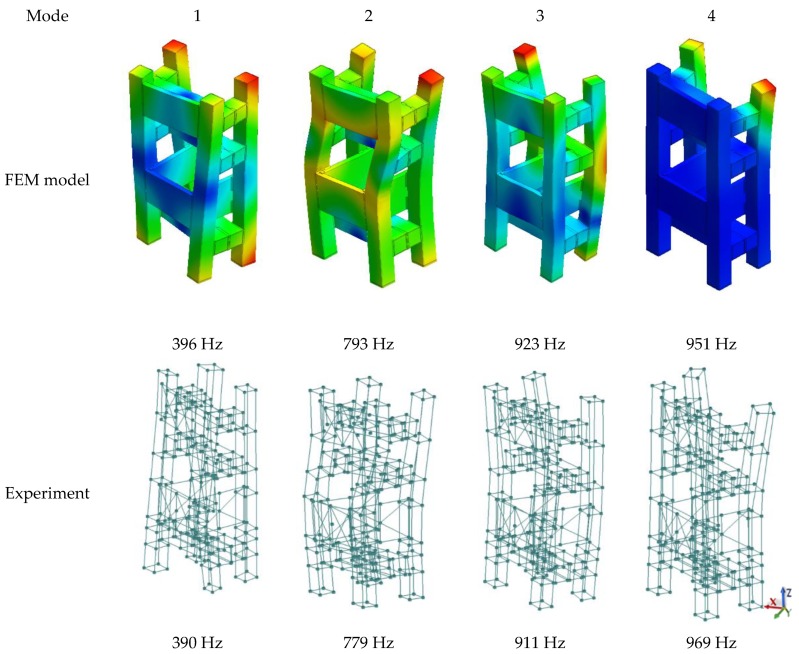
Comparison of selected calculated and experimentally determined mode shapes for the complex steel–polymer concrete frame.

**Figure 14:**
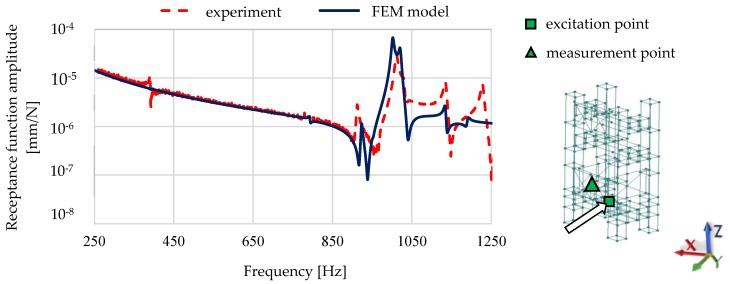
Comparison of the receptance functions calculated and experimentally determined for the complex steel–polymer concrete frame.

Selected mode shapes determined in the studied frequency range are shown in Figure 13, while the calculated and experimental receptance functions are shown in Figure 14.

## 4. Discussion

Summarizing the research presented in this article, the experimental studies show:

- The analysed beam meets the Maxwell’s Reciprocal Theorem, which indicates its linearity;
- The identity of the complex and real mode shapes were close, which indicates the proportional damping of the beam;
- The similar values of natural frequencies for forms 1 and 2, 3 and 4, and 6 and 7 may indicate the isotropicity of the polymer concrete;
- That, despite the highly heterogenous character of the polymer concrete used, the experimental mode shapes were similar to those of an isotropic homogenous beam, which may indicate the lack of mutual displacements between the steel profile and polymer concrete filling and that it can be modeled using a linear isotropic model of the material that is present in the composite structure.

Analytical studies showed that:

- The derived Hamiltonian Equation (3) indicates that it is possible to build a reliable analytical model of a composite steel–polymer concrete beam using an homogeneous beam model with equivalent bending stiffness $\left(E_{1}I_{1}+E_{2}I_{2}\right)$ and equivalent mass per unit length of the beam $\left(\rho_{1}A_{1}+\rho_{2}A_{2}\right)$;
- The analytical model could accurately represent mode shapes and natural frequencies of the analyzed beams: 50 mm × 50 mm (relative error below 4%, mean 3.1%), 70 mm × 70 mm (relative error below 2%, mean 0.47%) and 140 mm × 140 mm (relative error below 0.9%, mean 0.6%).

FEM studies showed that:

- The proposed FEM model of a steel–polymer concrete beam ensured agreement of the mode shapes with the experimentally determined mode shapes;
- The agreement of the mode shapes for torsional and flexural vibrations show the correctness of adopting the linear elastic isotropic model for polymer concrete; it confirms conclusions (i), (iii) and (iv);
- The application of the structural damping model, expressed as a complex stiffness model, enables a good representation of the damping properties of steel–polymer concrete beams, which is shown in the high compliance of frequency response functions (FRFs) calculated and experimentally determined; this also confirms conclusion (ii);
- The developed procedure and the determined model parameters allow for the development of reliable models for steel–polymer concrete beams with sizes: 50 mm × 50 mm (relative error below 3%, mean 1.4%), 70 mm × 70 mm (relative error below 5%, mean 2.6%) and 140 mm × 140 mm (relative error below 3%, mean 2.6%) for natural frequencies;
- The developed FEM model is a good alternative to continuous models, showing only slightly larger discrepancies compared to the results of experimental tests.
- The presented results of tests and analyses indicate that the proposed methodology of modeling the dynamic properties of steel–polymer concrete beams facilitates obtaining the results of calculations with high reliability, both in terms of quality and quantity, also for complex structures.

In particular:

- Agreement of the mode shape was obtained for the model of a complex steel–polymer concrete structure;
- Relative errors obtained by comparing the calculated and experimentally determined values did not exceed 3%, on average they were 1.7%, which proves good quantitative agreement of the model with the real structure;
- The calculated frequency response functions obtained for the complex model of a structure consisting of steel–polymer concrete beams showed good agreement with experimental data, which showed that the model reliably reproduced the dynamic properties of the structure in the studied frequency range;
- A reliable description of the dynamic properties of a complex steel–polymer concrete structure was obtained without additional (global) model updating.

## 5. Conclusions

The modeling method presented in this article has a large application potential in constituting a tool supporting the design of complex steel–polymer concrete structures. The conducted comprehensive research, i.e., experimental tests complemented with analysis of a mathematical models, helps us to understand the phenomena occurring in a complex steel–polymer concrete structure. Based on this understanding, it is possible to adopt simplifications that allow the building of a reliable, relatively simple finite element model which competes with the continuous models (described with differential equations) presented in the literature.

One of the key simplifications adopted is the use of a linear elastic isotropic material to model the highly heterogenous polymer concrete. This simplification reduced the model’s complexity by eliminating the necessity of modeling the polymer concrete as fine and coarse aggregates bound with an epoxy resin, and at the same time obtaining reliable results—the models presented in this paper are characterized by the agreement of the mode shapes, and relative errors in the case of natural frequencies below 5%, in relation to the results of experimental tests.

Another important feature of the presented model, distinguishing it from others presented in the literature review, is the high compliance of FRFs with the experimental data. The obtained FRFs enable the reliable assessment of dynamic properties of the analyzed structures across a wide frequency range, taking into account their damping properties. This is a key aspect in the process of designing mechanical structures, as it enables the determination of the real displacements occurring in the structure under the influence of external loads.

However, the presented method may have some limitations resulting from the adopted model assumptions. The first of these may be the use of polymer concrete, in which the binder is a resin with high shrinkage. It can cause contact loss on the steel–polymer concrete contact surface, contradicting one of the presented model assumptions. Another limitation may be the application of the presented method for modeling beams with a cross-section smaller than 50 mm × 50 mm, for a composition similar to the polymer concrete presented in the article. This limitation is due to the dimensions of the coarse fraction elements of the mineral filling (8–16 mm), and the associated possible poor mixing of the mineral filling, leading to a disruption of the structure of polymer concrete [40]. In this case, it may be necessary to scale the size of the mineral fill to get accurate modeling results.

In summary, the modeling methodology presented in this paper facilitates obtaining a reliable description of the dynamic properties of both steel–polymer concrete beams and the complex structures constructed from them.

## Figures and Tables

**Table 1:** Composition of the applied filling polymer concrete.

Component	Epoxy Resin	Ash	Fine Fraction (0.25–2 mm)	Medium Fraction (2–10 mm)	Coarse Fraction (8–16 mm)
Mass percentage	15%	1%	19%	15%	50%

**Table 2:** Parameters of signal acquisition.

Parameter	Value
Sampling rate	4096 Hz
Frequency resolution	0.5 Hz
Signal acquisition time	2 s
Frequency response function estimator	H_1_
Number of averages	10
Scaling of the frequency response function	global

**Table 3:** Material properties of the steel and polymer concrete determined experimentally.

Property	Steel	Polymer Concrete
Modulus of elasticity E	210 ± 5 GPa	17.2 ± 0.2 GPa
Poisson’s ratio ν	0.28 ± 0.03	0.20 ± 0.05
Density ρ	7812 ± 35 kg/m^3^	2200 ± 6 kg/m^3^
Loss factor η	0.00220 ± 0.00005	
Equivalent loss factor	0.00480 ± 0.00024	

**Table 4:** Parameters of the modal model of the steel–polymer concrete beam.

Mode Shape	Natural Frequency	Modal Damping
1.	339 Hz	0.16%
2.	341 Hz	0.25%
3.	899 Hz	0.16%
4.	900 Hz	0.13%
5.	1265 Hz	0.17%
6.	1665 Hz	0.16%
7.	1669 Hz	0.17%

**Table 5:** Experimental verification of the natural frequencies of the steel–polymer concrete beam model following the identification of its parameters.

Model	Experiment	FEM Model	Relative Error *δ_FEM_*	Continuous Model	Relative Error *δ_C_*
1.	339 Hz	343 Hz	~1.2%	338 Hz	<0.3%
2.	341 Hz	343 Hz	<0.6%	338 Hz	<0.9%
3.	899 Hz	928 Hz	~3.2%	899 Hz	~0%
4.	900 Hz	928 Hz	~3.1%	899 Hz	<0.1%
5.	1265 Hz	1266 Hz	~0%	1242 Hz	<2%
6.	1665 Hz	1755 Hz	~6.0%	1665 Hz	~0%
7.	1669 Hz	1755 Hz	~5.2%	1665 Hz	~0%

**Table 6:** Comparison of calculated and experimentally determined natural frequencies for 50 mm × 50 mm and 140 mm × 140 mm beams.

Mode Shape	Experiment	FEM Model	$\mathrm{Relative}\;\mathrm{Error}\;\delta_{FEM}$	Continuous Model	Relative Error *δ_C_*
50 mm × 50 mm beam					
1.	247 Hz	248 Hz	<0.1%	241 Hz	~2.4%
2.	251 Hz	248 Hz	~1.2%	241 Hz	<4.0%
3.	676 Hz	685 Hz	~1.3%	655 Hz	~3.1%
4.	678 Hz	685 Hz	~1.0%	655 Hz	~3.4%
5.	1276 Hz	1241 Hz	~2.7%	1243 Hz	~2.6%
140 mm × 140 mm beam					
1.	628 Hz	648 Hz	~3.2%	630 Hz	~0.3%
2.	629 Hz	648 Hz	~3.0%	630 Hz	~0.2%
3.	1252 Hz	1265 Hz	~1.0%	1242 Hz	~0.8%
4.	1526 Hz	1567 Hz	<2.7%	1539 Hz	~0.9%
5.	1527 Hz	1567 Hz	<2.6%	1539 Hz	~0.8%

**Table 7:** Comparison of the values of the calculated and experimental natural frequencies of the steel–polymer concrete frame.

Mode Shape	FEM Model	Experiment	Relative Error δ
1.	396 Hz	390 Hz	<2%
2.	793 Hz	779 Hz	<2%
3.	923 Hz	911 Hz	~1%
4.	951 Hz	969 Hz	<2%
5.	1002 Hz	1012 Hz	<1%
6.	1021 Hz	1031 Hz	<1%
7.	1135 Hz	1135 Hz	<1%
8.	1190 Hz	1228 Hz	~3%
9.	1314 Hz	1284 Hz	~2%

## List of Used Symbols

$A_{i}$	Cross-section area ($m^{2}$)
$E_{i}$	Young’s modulus (Pa)
$G_{i}$	Shear modulus (Pa)
$H_{i}$	Outer height of the beam cross-section (m)
$h_{i}$	Inner height of the beam cross-section (m)
$I_{i}$	Area moment of inertia ($m^{4}$)
$I_{pi}$	Polar moment of inertia ($m^{4}$)
i	Material: steel (1), polymer concrete (2)
j	Imaginary unit
k	Shear correction factor
$K_{i}$	Torsional parameter ($m^{2}$)
L	Length of a beam (m)
w	Transverse deflection (m)
ρ	Mass density $\left[\frac{\mathrm{kg}}{m^{3}}\right]$
